# Application of Fused Reality Holographic Image and Navigation Technology in the Puncture Treatment of Hypertensive Intracerebral Hemorrhage

**DOI:** 10.3389/fnins.2022.850179

**Published:** 2022-03-11

**Authors:** Chen Peng, Liu Yang, Wang Yi, Liang Yidan, Wang Yanglingxi, Zhang Qingtao, Tang Xiaoyong, Yongbing Tang, Wang Jia, Yu Xing, Zhu Zhiqin, Deng Yongbing

**Affiliations:** ^1^Department of Neurosurgery, Chongqing Emergency Medical Center, Chongqing University Central Hospital, Chongqing, China; ^2^QINYING Technology Co., Ltd., Chongqing, China; ^3^College of Automation, Chongqing University of Posts and Telecommunications, Chongqing, China

**Keywords:** hypertensive intracerebral hemorrhage, minimally invasive puncture and drainage, mixed reality, navigation, deviation

## Abstract

**Objective:**

Minimally invasive puncture and drainage (MIPD) of hematomas was the preferred option for appropriate patients with hypertensive intracerebral hemorrhage (HICH). The goal of our research was to introduce the MIPD surgery using mixed reality holographic navigation technology (MRHNT).

**Method:**

We provided the complete workflow for hematoma puncture using MRHNT included three-dimensional model reconstruction by preoperative CT examination, puncture trajectory design, immersive presentation of model, and real environment and hematoma puncture using dual-plane navigation by wearing special equipment. We collected clinical data on eight patients with HICH who underwent MIPD using MRHNT from March 2021 to August 2021, including the hematoma evacuation rate, operation time, deviation in drainage tube target, postoperative complications, and 2-week postoperative GCS.

**Result:**

The workflow for hematoma puncture using MRHNT were performed in all eight cases, in which the average hematoma evacuation rate was 47.36±9.16%, the average operation time was 82.14±15.74 min, and the average deviation of the drainage tube target was 5.76±0.80 mm. There was no delayed bleeding, acute ischemic stroke, intracranial infection, or epilepsy 2 weeks after surgery. The 2-week postoperative GCS was improved compared with the preoperative GCS.

**Conclusion:**

The research concluded it was feasible to perform the MIPD by MRHNT on patients with HICH. The risk of general anesthesia and highly professional holographic information processing restricted the promotion of the technology, it was necessary for technical innovation and the accumulation of more case experience and verification of its superiority.

## 1. Introduction

Stroke has become the leading cause of death in China. Hypertensive intracerebral hemorrhage (HICH) is one of the most serious complications of hypertension, with an incidence of 19–48% of strokes in China, and the high disability and mortality rates of HICH lead to a heavy social burden (Zhou et al., 2019). At present, there is no evidence for the optimal surgical treatment of HICH with surgical indications. MISTIE research demonstrated the safety profile of the minimal invasive surgery procedure revealed clot size reduction could be achieved with similar safety to standard medical treatment (Hanley et al., 2016, 2019).

The precise puncture of hematomas is the key to the success of surgeries, and the methods used include the “blind” method, which uses a freehand technique according to CT images combined with skull anatomical marks, CT-guided (Wang et al., 2009) and image-guided (Yang et al., 2014; Sun et al., 2016) puncture methods and the neuronavigation system (Chartrain et al., 2018) puncture method. However, all the above puncture methods have shortcomings, such as inaccuracy, expensive, non-portable, bulky hardware. It is important to find a more convenient, visualized, rapid, and precise puncture method.

Mixed reality has been developed based on virtual reality and augmented reality technologies. By processing holographic images, mixed reality provides virtual images and information in the real environment and provides users with immersive feelings. Users can obtain real and virtual image information at the same time by wearing special equipment (Microsoft, HoloLens) and interact with holographic images in the display environment according to their own commands. With this technology, neurosurgeons can first construct intracerebral hemorrhages and design the puncture trajectory. During surgery, the location and morphology of a hematoma can be observed from multiple angles, and precise puncture can be performed with the help of navigation.

Several studies on glioma, meningioma, intracranial aneurysm have shown that MR technology could implement a safe, effective, and minimally invasive individualized operation plan, evaluate the operation risk, and protect the tissue structure during the operation (Kockro et al., 2016; Incekara et al., 2018; Qi et al., 2021; Zhang et al., 2021). There are no reports on the application of MR technology to hematoma puncture in patients with HICH. In this research, we introduce the MIPD surgery using mixed reality holographic navigation technology (MRHNT). We provide the complete workflow, show the clinical data and results, shar our practical experience in hematoma puncture using MRHNT, and verify the accuracy and feasibility of the application of this technology.

In this research, we introduced a precise MIPD method in different parts of HICHs using mixed reality holographic navigation technology (MRHNT). We provided the complete workflow, showed the clinical data and results, shared our practical experience in hematoma puncture using MRHNT, and verified the accuracy and feasibility of the application of this technology.

## 2. Materials and Methods

### 2.1. Clinical Datae

From March 2021 to August 2021, approved by the ethics committee of Chongqing Emergency Medical Center, HICH patients treated with MIPD by mixed reality holographic navigation technology were involved in this research. All patients signed the surgical informed consent form. Partially in accordance with the MISTIE study the inclusion criteria were following: patients with non-traumatic (spontaneous) ICH not due to a macrovascular cause such as an aneurysm or AVM were involved. All patients signed the surgical informed consent form. All patients age was 18–80 years old with GCS score ≥14 or NIHSS score ≤ 6, whose ICH remained the same size for at least 6 h after diagnostic CT. Our surgery involved patients with both supratentorial and supratentorial hemorrhage, with supratentorial hematoma volume of 30–50 ml, cerebellar hematoma volume of 10–15 ml, with brainstem hematoma volume of 5–10 ml. The exclusion criteria were as follows: patients with cerebral herniation due to HICH, severe cardiopulmonary disease, or coagulopathy, other patients who cannot tolerate general anesthesia, and patients with family members who refused surgery by mixed reality holographic navigation technology. We analyzed eight patients based on their preoperative and postoperative hematoma volume, hematoma evacuation rate, operation time, blood loss, deviation in drainage tube target (the distance between the tip of the drainage tube and the designed puncture trajectory target), 2-week rebleeding rate4, postoperative complications, and preoperative and 2-week post-operative GCS.

### 2.2. Preoperative CT Examination and Design Puncture Trajectory

All patients were required to undergo head CT examination before the operation. Patients were examined by placing three sticky analysis markers around the puncture area. After anesthesia, a bone nail was drilled through the hole in the sticky marker base, and a sterilized analysis marker was placed; these two markers were the “twin marker” and ensured no obvious deviation in the location of the markers. CT data were collected by a 64-slice CT scanner (Lightspeed VCT 6, General Electric Company, USA). Image parameters included exposure (3 mAS), thickness (5 mm), and image size (512,512). Based on hospital PACS, DICOM format data were imported into Medical Modeling and Design System software for reconstruction of the head model. Three-dimensional reconstructions were focused on the skull, hematoma, nose, and ears during head model building. Preoperative hematoma volume was measured by Medical Modeling and Design System software. According to the reconstructed head model, the puncture skull location and the hematoma target were planned to design a puncture trajectory. The designed puncture trajectory has the same diameter as the actual puncture needle. The depth of the designed puncture trajectory was also measured.

### 2.3. Registration of Holographic Images

After anesthesia, three-dimensional coordinate locations data of the calibration plate, puncture needle, and three markers in head were captured by camera. We matched the preoperative reconstructed head mode with the coordinate data by MAYA software, bond location of the corresponding skull, hematoma, nose, and ears by analysis markers and imported the matched information into Microsoft HoloLens. The camera captured dynamic changes in the analysis marker location of the head and puncture needle, synchronizing holographic models with tracking software. This procedure took ~40 min.

### 2.4. Dual-Plane Navigation Puncture

**Figure 1:**
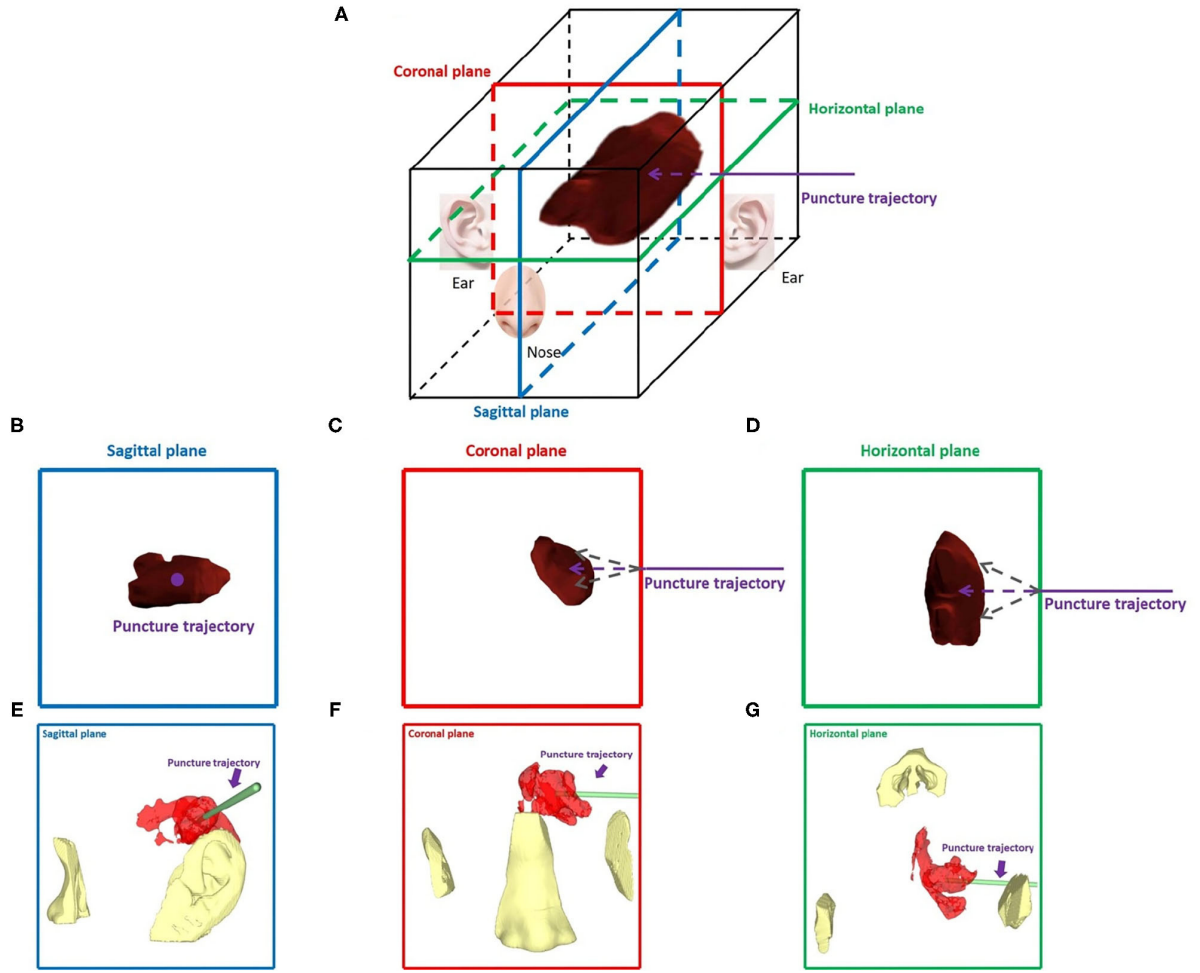
Dual-plane navigation puncture. **(A)** The head was considered a six-sided cube with horizontal, sagittal, and coronal planes. According to the hematoma puncture trajectory designed before surgery, the puncture angle and depth were observed from two planes. **(B–G)** For example, a hematoma was punctured at the basal ganglia from the temporal region, and hematoma and puncture trajectories were observed in the sagittal, coronal, and horizontal planes, respectively. **(B–D)** Theoretical images of different planes. **(E–G)** Wearing HoloLens, images of the different planes were presented by adjusting the locations of the nose and ear.

Innovatively, since a double-arm digital subtraction angiography device can observe vascular morphology from two angles, we considered the head to be a six-sided cube with horizontal, sagittal, and coronal planes. If the puncture trajectory was perpendicular to a plane, the other two planes could be observed to evaluate the deviation in the puncture trajectory from the horizontal and vertical directions. For example, when puncturing a hematoma at the basal ganglia from the temporal region, the puncture trajectory was perpendicular to the sagittal plane, and we observed the vertical and horizontal deviation between the puncture needle and the designed puncture trajectory from the coronal plane and horizontal plane. When wearing mixed reality holographic equipment, the three-dimensional sense of the space will be more obvious. According to the locations of the nose and ear, gestures such as rotation and movement were used to adjust the plane angle and location, and then, the image was locked. After the image was locked, the holographic image could not change due to gestures, making this method more convenient for the puncture operation of both hands. We illustrated this method in Figure 1.

### 2.5. Surgical Procedure

**Figure 2:**
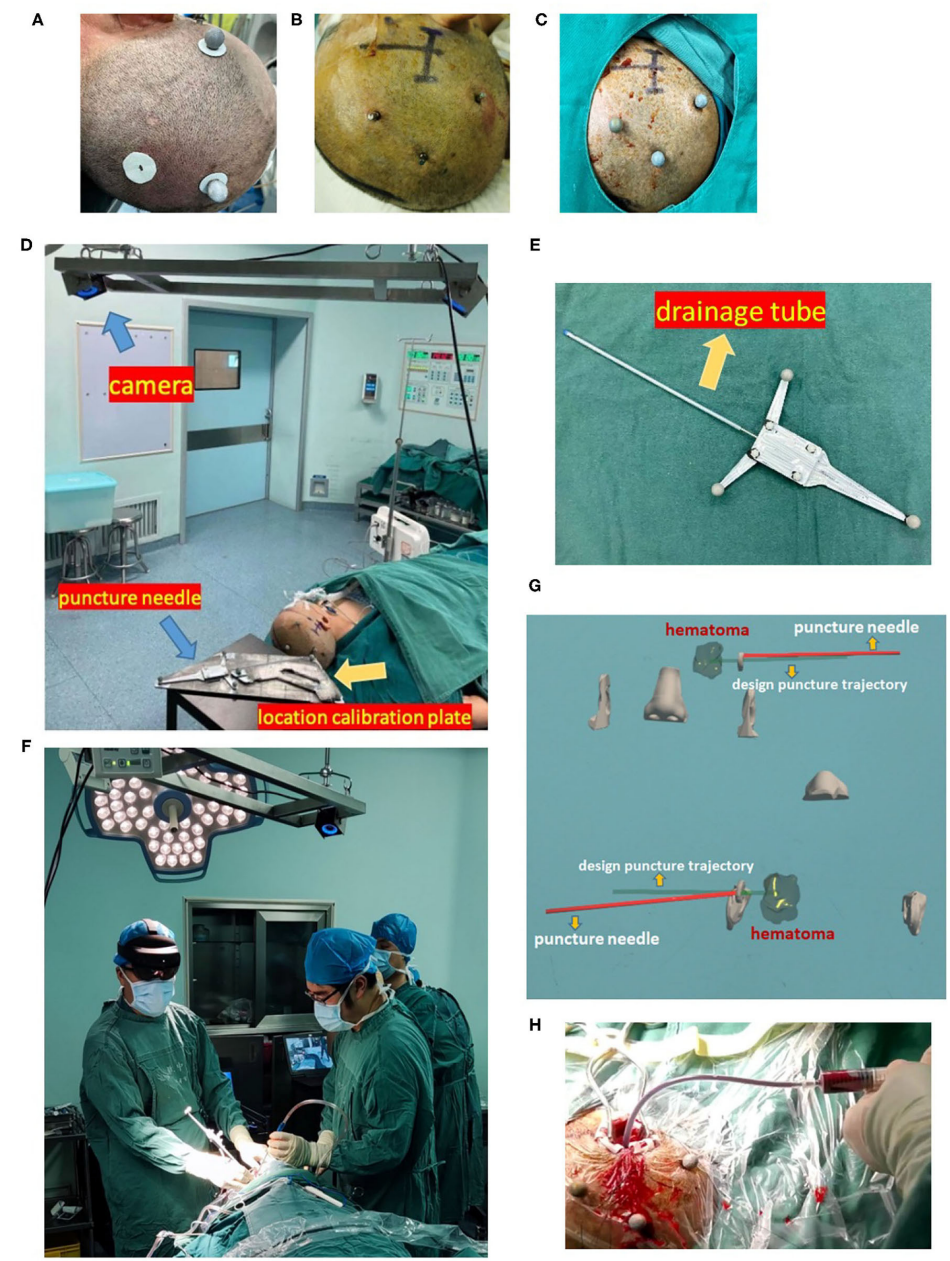
Workflow of the minimally invasive puncture and drainage of hypertensive intracerebral hemorrhages by mixed reality holographic navigation technology. **(A)** Patients wore three sticky analysis markers around the puncture area. **(B)** A bone nail was drilled through the hole in the sticky marker base, which was replaced with sticky analysis markers, and these “twin marker” ensured no obvious deviation in the location of markers. **(C)** After disinfection, a sterilized analysis marker was installed on the bone nail. **(D)** Three-dimensional coordinate data of the location calibration plate, puncture needle, and head were captured by a camera. **(E)** Combination of a puncture needle and drainage tube. **(F)** Wearing HoloLens, the surgeon used MRHNT for hematoma puncture. **(G)** Wearing HoloLens, the surgeon actually viewed the two planes of the image. **(H)** After hematoma puncture was completed, the hematoma was aspirated from the drainage tube.

The doctor wore mixed reality holographic equipment to observe the precise locations of the skull, hematoma, nose, and ears, designed puncture trajectory and actual puncture needle. According to the designed puncture trajectory, we performed skin incision and skull drilling, performed hematoma puncture according to the above dual-plane navigation puncture technology, aspirated the hematoma, retained the drainage tube, and sutured the skin. When the operator observed the puncture needle entering the hematoma target, removed the puncture needle, retained drainage tube, and connected with a 10 ml syringe to aspirate until there was no longer any fluid component of the clot. The drainage tube was tunneled subcutaneously, and connected to closed drainage system. We performed postoperative head CT examination, but did not inject rtPA or other drugs in the drainage tube as in the MISTIE study, and kept the drainage tube in low drainage for 48 h and then removed it. We provide the complete workflow of this technology in Figure 2.

### 2.6. Follow-Up Imaging and Accuracy Assessment

Head CT examination was performed immediately or 1 day after surgery. Postoperative hematoma volume was measured by a non-operator, as described above. Hematoma evacuation rate = (pre-operative hematoma volume- post-operative hematoma volume)/pre-operative hematoma volume. Accuracy assessment was defined as the deviation between the drainage tube and the planned puncture hematoma target. The deviation calculation used BLENDER2.93.3 software, which used the 3D XYZ coordinate system to visualize the deviation between the drainage tube and the target point (points 0, 0, and 0).

### 2.7. Statistical Analysis

Quantitative data were presented as means ± SDs. The paired *t*-test was used to compare the difference between the preoperative and postoperative hematoma volumes and GCS. All statistical analyses were performed using SPSS version 21 (IBM SPSS Statistics for Macintosh, IBM Corp). In all cases, a *p* < 0.05 was considered statistically significant.

## 3. Results

From March 2021 to August 2021,8 patients with HICHs were treated with MIPD by mixed reality holographic navigation technology, including five males and three females with an average age of 52.5±13.42 years (range, 37–69 years). The hematoma was located in the basal ganglia in five cases, in the brainstem in two cases and in the temporal lobe in one case. Among six patients with supratentorial hematoma, four cases of postoperative hematoma were more than 15 ml, and 1 case was more than 20 ml, with the average post-operative hematoma was 17.3 ml. The average hematoma evacuation rate in eight patients was 47.36 ± 9.16 %. There were statistically significant differences in the pre-operative and post-operative hematoma (*P* = 0.002) volumes. All operations were performed under general anesthesia, the average operation time was 82.14±15.74 min, and the average intraoperative blood loss was 36.28±8.14 ml. By double-plane MRHNT, the average deviation in the drainage tube target was 5.76±0.80 mm. There was no delayed bleeding, acute ischemic stroke, intracranial infection, or epilepsy 2 weeks after surgery. The average preoperative GCS was 9.25±2.05, while the 2-week postoperative GCS was 11.00±2.39. The 2-week postoperative GCS was improved, but it was not statistically significant (*P* = 0.26) compared with the preoperative GCS. A summary of demographic and clinical characteristics is provided in Table 1.

**Table 1:** Demographic and clinical data of eight patients in the research.

**Cases**	**Age (years), Gender**	**Hematoma location**	**Pre-operative volume (ml)**	**Post-operative volume (ml)**	**HER (%)**	**Deviation (mm)**
1	69, M	Temporal lobe	32.17	17.23	46.44	7.08
2	47, M	Basal ganglia	33.10	12.48	62.30	5.62
3	37, M	Brainstem	5.45	3.18	41.65	4.22
4	69, M	Basal ganglia	30.34	18.81	38.00	6.04
5	43, M	Basal ganglia	31.69	19.41	38.75	5.46
6	44, M	Basal ganglia	37.22	15.32	58.84	5.87
7	44, M	Brainstem	6.32	3.12	50.63	6.13
8	67, F	Basal ganglia	35.22	20.34	42.25	5.66

## 4. Technology Advantages

**Figure 3:**
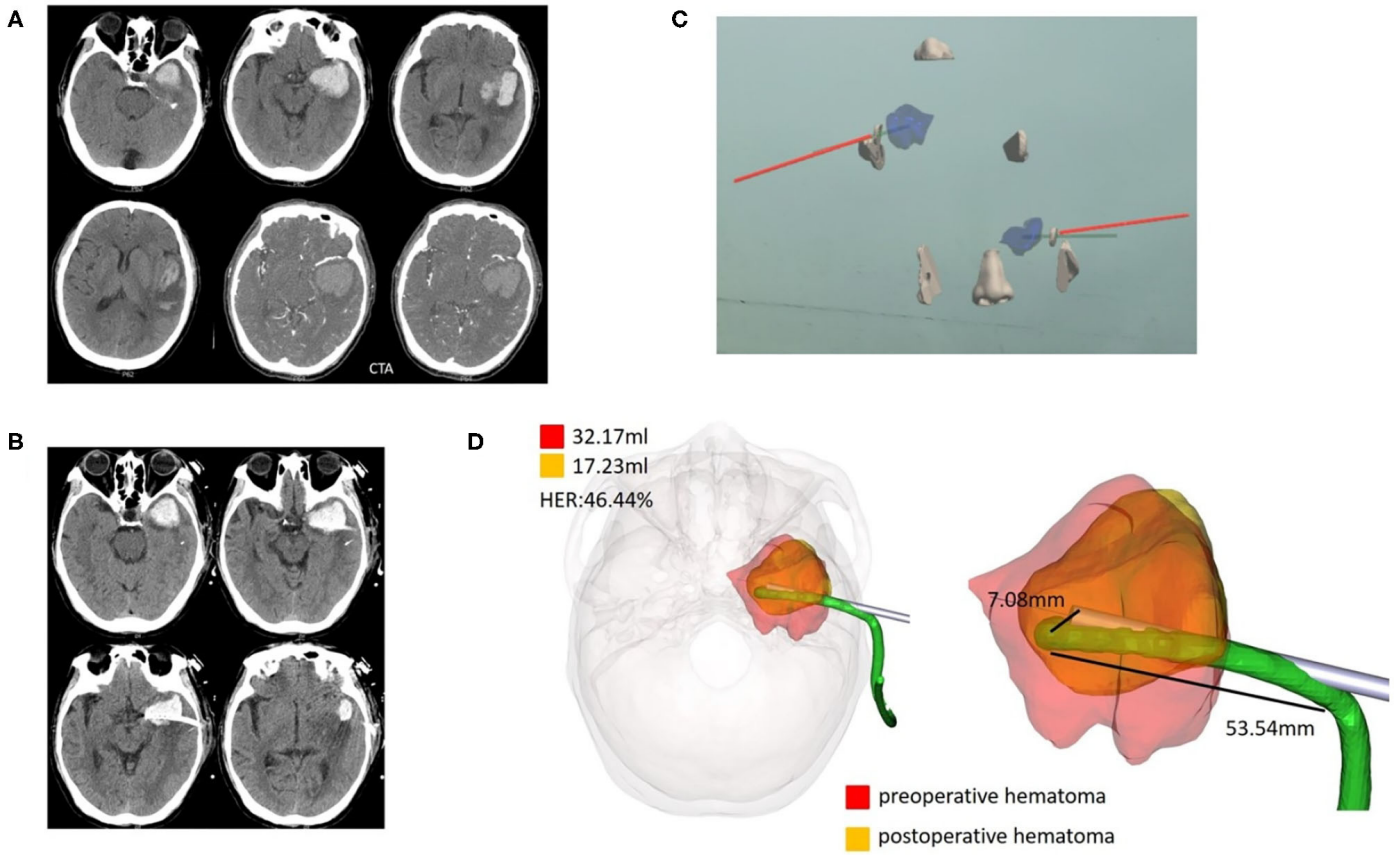
Case 1, a 69-year-old male patient diagnosed with HICH in the left temporal lobe. **(A)** Preoperative CT showed a HICH in the left temporal lobe, excluding aneurysms, and arteriovenous malformation. **(B)** Postoperative follow-up CT. **(C)** Wearing HoloLens, the coronal, and horizontal planes were adjusted for puncture through the ear and nose locations. **(D)** For fusion of the preoperative and postoperative three-dimensional reconstruction of hematomas, the preoperative hematoma volume was 32.17 ml, the postoperative hematoma volume was 17.23 ml, and the hematoma evacuation rate was 46.44%. The length of the intracranial drainage tube was 53.54 mm, and the deviation in the drainage tube target was 7.08 mm. HER, hematoma evacuation rate.

**Figure 4:**
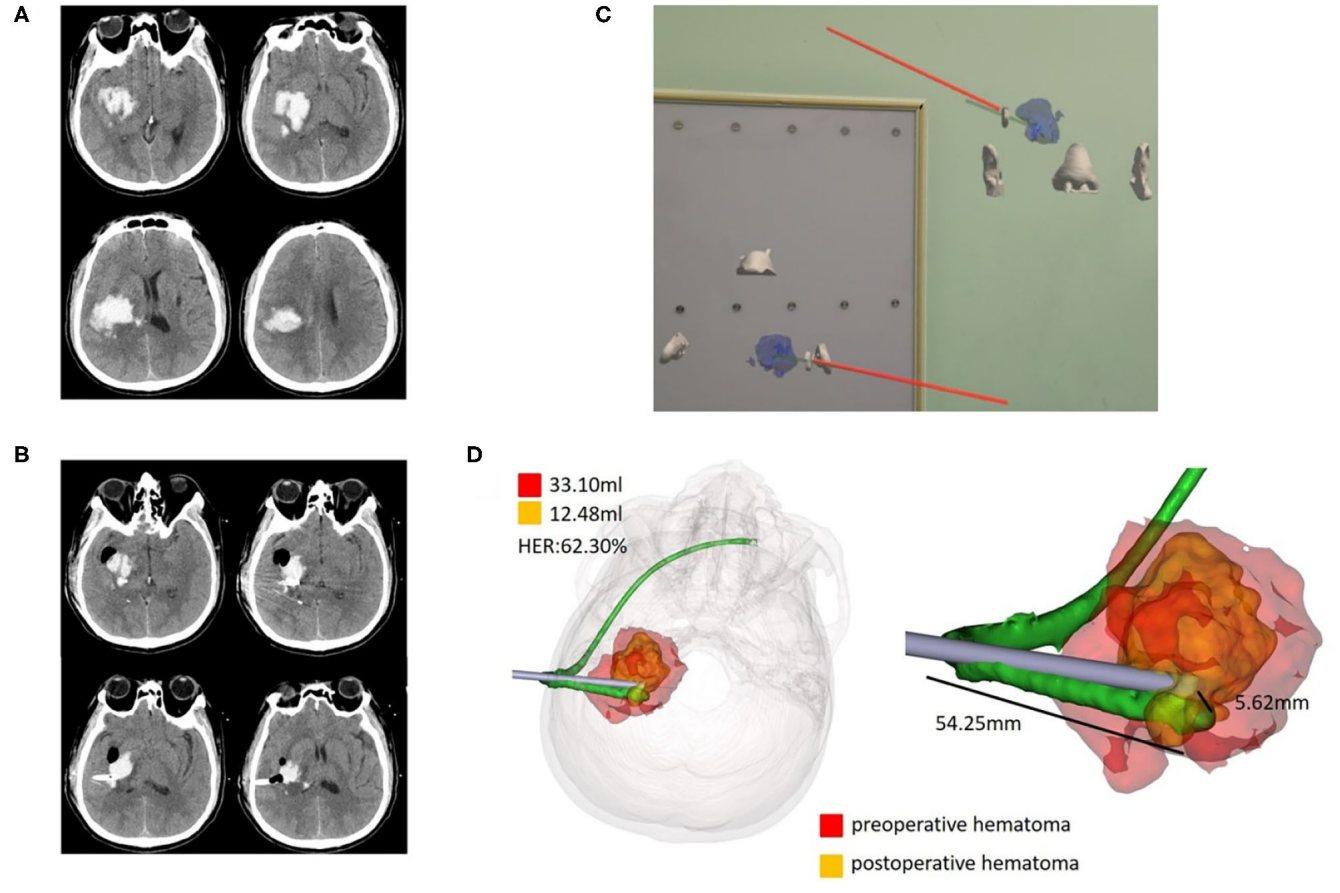
Case 2, a 47-year-old male patient diagnosed with HICH in the right basal ganglia. **(A)** Preoperative CT showed HICH in the right basal ganglia. **(B)** Postoperative follow-up CT. **(C)** Wearing HoloLens, adjust the coronal, and horizontal planes for puncture through the ear and nose location. **(D)** Fusion of the preoperative and postoperative three-dimensional reconstructions of the hematoma. The preoperative hematoma volume was 33.10 ml, the postoperative hematoma volume was 12.48 ml, and the hematoma evacuation rate was 62.30%. The length of the intracranial drainage tube was 54.25 mm, and the deviation of the drainage tube target was 5.62 mm. HER, hematoma evacuation rate.

**Figure 5:**
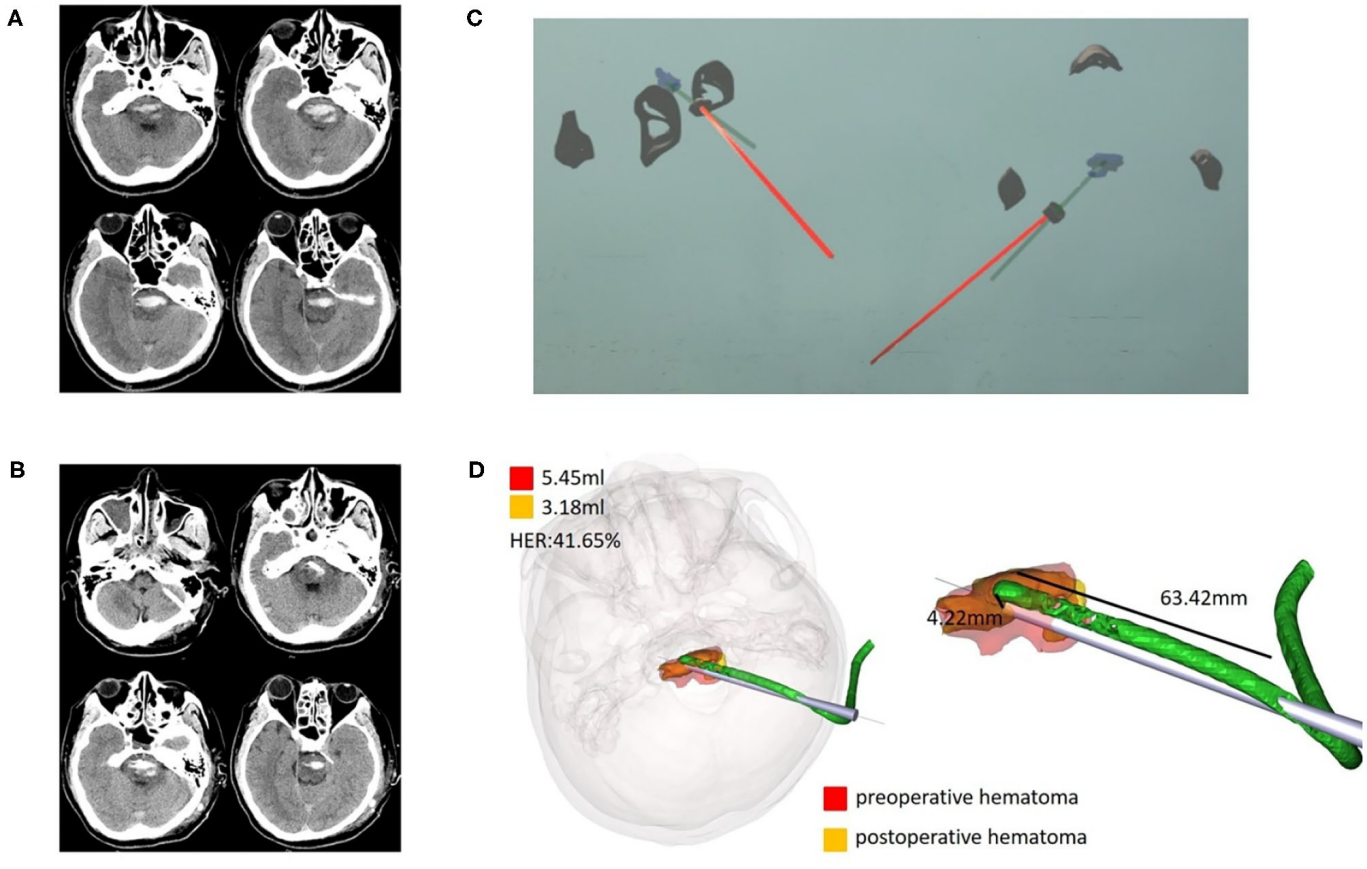
Case 3, a 37-year-old male patient diagnosed with HICH in the brainstem. **(A)** Preoperative CT showed HICH in the brainstem. **(B)** Postoperative follow-up CT. **(C)** Wearing HoloLens, the sagittal and horizontal planes were adjusted for puncture through the ear and nose locations. **(D)** Fusion of the preoperative and postoperative three-dimensional reconstructions of the hematoma. The preoperative hematoma volume was 5.45 ml, the postoperative hematoma volume was 3.18 ml, and the hematoma evacuation rate was 41.65%. The length of the intracranial drainage tube was 63.42 mm, and the deviation in the drainage tube target was 4.22 mm. HER, hematoma evacuation rate.

Wearing sticky analysis markers for preoperative CT examination could greatly shorten the time for holographic image registration. Mixed reality holographic image technology succeeded in creating stereoscopic sensations of the skull, hematoma, designed puncture trajectory, and actual puncture needle, and this immersive holographic environment was difficult to fully express through photos or videos or even AR technology. In addition, mixed reality holographic images were transferred to the screen in real time, allowing observers without experience to share the same view with the surgeon. Innovatively, dual-plane navigation puncture technology was used with camera monitoring, which allowed the head to move. After adjusting the two observation puncture planes by gestures, the holographic image was locked to avoid image changes caused by gestures. We used both hands to hold the head and tail of the puncture needle to enhance the stability of the puncture. We observed the puncture direction with dynamic navigation from two planes to better control the puncture deviation. We obtained a puncture deviation of 5.76±0.80 mm, which was perfectly acceptable for a hematoma volume of ~30 ml. Representative cases are presented in Figures 3–5.

## 5. Discussion

In patients with HICH, MIPD of hematomas was the preferred option for appropriate patients. Surgery first required the localization of the hematoma, whereas hematomas in HICH did not require millimeter accuracy, and experienced neurosurgeons could successfully puncture the hematoma with various localization methods. Stereotactic devices and neuronavigation systems seemed overqualified for hematoma localization and were not available in many hospitals. Additionally, neuronavigation system also has the disadvantages of expensive, non-portable, and with bulky hardware. However, various puncture methods mainly rely on personal experience, and disadvantages include poor accuracy, a high failure rate, and difficulty in ensuring the homogeneity of the puncture location, which affects the surgical effect and increases the surgical risk.

Mixed reality technology integrates holographic image information into the real world by a computer. The real environment and virtual images could be spliced in the same field of view in real time for a three-dimensional display. Owing to advancements in holographic information transmission and processing. Our solution of using professional software, we take CT image and realistic environment information captured by camera to reconstruct, match, and generate holographic images. The method did not require additional cost or technical complexity, other than few professional software. This technology rendered MRHNT as much more convenient, affordable, portable, and popular.

We acquired head information using the technology to achieve holographic image and real head integration. By wearing mixed reality holographic equipment, we could understand the precise location of intracranial hematomas, tumors, ventricles, and other structures with the perspective function, which laid a theoretical foundation for implementation in neurosurgery. This technology should have a promising future in medicine, but it is still in its infancy and is in the initial stage; its application in neurosurgery has rarely been reported (Zhang et al., 2021).

In the preliminary work, our team rigidly matched the holographic image processed by the computer with the head, visualized the ventricular structure, intuitively guided ventricular puncture operation, and improved the puncture accuracy compared with that of the traditional method. Moreover, mixed reality technology played a very helpful role in finding foreign bodies and locating hematomas in patients with traumatic brain injury. We reported a case of the localization of the intracranial nail and hematoma by mixed reality technology, which helped us to design the surgical incision rationally and avoid secondary injury caused by blind exploration (Li et al., 2018, 2021).

There were obvious shortcomings in the previous method, including low registration speed and rigid integration of the hologram to the head to avoid movement of the head. In particular, preliminary technology was not truly navigational; when the puncture needle was drilled into the skull, it could not be tracked.

To solve the above problems, we made several improvements as follows. First, patients wore three sticky analysis markers for head CT examination before the operation. We replaced sticky analysis markers with “twin marker” to ensure no obvious deviation in the location of markers. Then, the three-dimensional coordinate data were captured by a camera, which could shorten the holographic image registration time. Second, we abandoned the rigid matching of the holographic image and head by the eye. Alternatively, the camera captured dynamic changes in the analysis marker location of the head and puncture needle, synchronizing holographic images. This meant that even if the head location changed, the holographic image would change accordingly by analyzing the marker space distance through the camera. Third, we observed the puncture direction from dynamic navigation from the two planes to better control puncture deviation.

The results of eight patients with HICHs treated with MIPD by mixed reality holographic navigation technology revealed that the operation time and blood loss were acceptable. The hematoma evacuation rate was 47.36±9.16%, the average of supratentorial postoperative hematoma volume in six patients in our research was 17.3 ml. According to the results of the MISTIE study revealed reduction in clot size to 15 ml or less was associated with functional improvement. Although GCS score improved 2 weeks after surgery, this result was not comparable with MISTIE study, considering the small number of cases, not much preoperative hematoma volume (30–40 ml), and the absence of control group.

At present, most precise and popular of the previous methods were neuronavigation systems. van Doormaal et al. (2019) reported compared to the mean fiducial registration error of conventional neuronavigation was 3.6 mm, the mean fiducial registration error of holographic neuronavigation was 4.4 mm in three patients. Other researches have shown that the navigation deviation using mixed reality holographic navigation was 4–6 mm (Incekara et al., 2018; Li et al., 2018; McJunkin et al., 2018), which was consistent with our results. We obtained a puncture deviation of 5.76 ± 0.80 mm, which was perfectly acceptable for a hematoma volume of ~30 ml.

To improve the puncture accuracy, we have the following suggestions: 1. When drilling the skull, try to drill a larger hole, and make the puncture needle coincide exactly with the designed puncture trajectory. 2. Hold the head and tail of the puncture needle with both hands, and adjust the puncture direction horizontally and vertically at any time. 3. Mark the puncture needle with depth, and determine the puncture depth by holographic image navigation.

Our research has some limitations. At present, we have relatively few cases, so there are not enough data to verify the advancement of the technology. The new technology bears the risk of general anesthesia and takes a long time for surgery, which might make many surgeons relatively apathetic about this technology. Improving the accuracy of the puncture also requires the surgeon to spend much time in the model for consistent practice.

We believe that as science and technology drive the accelerated progress of medicine, surgical procedure will be more simplified, and new equipment and methods will be developed to improve puncture accuracy. Fusion with MRI images with white matter cellulose information to design the optimal puncture trajectory (Liu et al., 2020; Zheng et al., 2020; Zhu et al., 2021), and accumulation of more cases experience and verification of its superiority.

## 6. Conclusion

With MRHNT, neurosurgeons can first construct three-dimensional model and design the puncture trajectory. During surgery, the location and morphology of the hematoma can be observed from multiple angles, and precise puncture can be performed with the help of dual-plane navigation. The risk of general anesthesia and highly professional holographic information processing restrict the promotion of the technology, it is necessary for technical innovation and the accumulation of more case experience and verification of its superiority.

## Data Availability Statement

The original contributions presented in the study are included in the article/supplementary material, further inquiries can be directed to the corresponding author/s.

## Ethics Statement

The studies involving human participants were reviewed and approved by Ethics Committee of Chongqing Emergency Medical Center. The patients/participants provided their written informed consent to participate in this study.

## Author Contributions

DY and CP participated in the design and coordination of the study and drafted the manuscript. LYi and WYa participated in the clinical evaluation of the patients. WYi and ZZ interpreted data reconstruction and image fusion. CP, LYa, TX, and ZQ performed the minimally invasive surgery. WJ and YX performed the statistical analysis. All authors read and approved the final manuscript.

## Funding

This study was financially supported by the Medical Research Project of Science and Technology Bureau Joint Health Commission, Chongqing, China (2020MSXM078).

## Conflict of Interest

WYi was employed by QINYING Technology Co., Ltd. The remaining authors declare that the research was conducted in the absence of any commercial or financial relationships that could be construed as a potential conflict of interest.

## Publisher's Note

All claims expressed in this article are solely those of the authors and do not necessarily represent those of their affiliated organizations, or those of the publisher, the editors and the reviewers. Any product that may be evaluated in this article, or claim that may be made by its manufacturer, is not guaranteed or endorsed by the publisher.

## References

[B1] Chartrain A. G. Kellner C. P. Fargen K. M. Spiotta A. M. Chesler D. A. Fiorella D.. (2018). A review and comparison of three neuronavigation systems for minimally invasive intracerebral hemorrhage evacuation. J. Neurointervent. Surg. 10, 66–74. 10.1136/neurintsurg-2017-013091 28710083

[B2] Hanley D. F. Thompson R. E. Muschelli J. Rosenblum M. McBee N. Lane K.. (2016). Safety and efficacy of minimally invasive surgery plus alteplase in intracerebral haemorrhage evacuation (MISTIE): a randomised, controlled, open-label, phase 2 trial. Lancet Neurol. 15, 1228–1237. 10.1016/S1474-4422(16)30234-4 27751554 PMC5154627

[B3] Hanley D. F. Thompson R. E. Rosenblum M. Yenokyan G. Lane K. McBee N.. (2019). Efficacy and safety of minimally invasive surgery with thrombolysis in intracerebral haemorrhage evacuation (MISTIE III): a randomised, controlled, open-label, blinded endpoint phase 3 trial. Lancet 393, 1021–1032. 10.1016/S0140-6736(19)30195-3 30739747 PMC6894906

[B4] Incekara F. Smits M. Dirven C. Vincent A. (2018). Clinical feasibility of a wearable mixed-reality device in neurosurgery. World Neurosurg. 118, e422–e427. 10.1016/j.wneu.2018.06.208 30257298

[B5] Kockro R. A. Killeen T. Ayyad A. Glaser M. Stadie A. Reisch R.. (2016). Aneurysm surgery with preoperative three-dimensional planning in a virtual reality environment: technique and outcome analysis. World Neurosurg. 96, 489–499. 10.1016/j.wneu.2016.08.124 27609450

[B6] Li Y. Chen X. Wang N. Zhang W. Li D. Zhang L.. (2018). A wearable mixed-reality holographic computer for guiding external ventricular drain insertion at the bedside. J. Neurosurg. 131, 1599–1606. 10.3171/2018.4.JNS18124 30485188

[B7] Li Y. Huang J. Huang T. Tang J. Zhang W. Xu W.. (2021). Wearable mixed-reality holographic navigation guiding the management of penetrating intracranial injury caused by a nail. J. Digit. Imaging 34, 362–366. 10.1007/s10278-021-00436-3 33846887 PMC8289971

[B8] Liu Y. Wang L. Cheng J. Li C. Chen X. (2020). Multi-focus image fusion: a survey of the state of the art. Inform. Fus. 64, 71–91. 10.1016/j.inffus.2020.06.013

[B9] McJunkin J. Jiramongkolchai P. Chung W. Southworth M. Durakovic N. Buchman C.. (2018). Development of a mixed reality platform for lateral skull base anatomy. Otol. Neurotol. 39, e1137–e1142. 10.1097/MAO.0000000000001995 30239435 PMC6242747

[B10] Qi Z. Li Y. Xu X. Zhang J. Li F. Gan Z.-C.. (2021). Holographic mixed-reality neuronavigation with a head-mounted device: technical feasibility and clinical application. Neurosurg. Focus 51:E22. 10.3171/2021.5.FOCUS21175 34333462

[B11] Sun G.-C. Chen X.-l. Hou Y.-Z. Yu X.-G. Ma X.-D. Liu G.. (2016). Image-guided endoscopic surgery for spontaneous supratentorial intracerebral hematoma. J. Neurosurg. 127, 537–542. 10.3171/2016.7.JNS16932 27636179

[B12] van Doormaal T. P. C. van Doormaal J. A. M. Mensink T. (2019). Clinical accuracy of holographic navigation using point-based registration on augmented-reality glasses. Oper. Neurosurg. 17, 588–593. 10.1093/ons/opz094 31081883 PMC6995446

[B13] Wang W.-Z. Jiang B. Liu G.-M. Li D. Lu C.-Z. Zhao Y.-D.. (2009). Minimally invasive craniopuncture therapy vs. conservative treatment for spontaneous intracerebral hemorrhage: results from a randomized clinical trial in china. Int. J. Stroke 4, 11–16. 10.1111/j.1747-4949.2009.00239.x 19236490

[B14] Yang Z. Hong B. Jia Z. Chen J. Ge J. Han J.. (2014). Treatment of supratentorial spontaneous intracerebral hemorrhage using image-guided minimally invasive surgery: initial experiences of a flat detector ct-based puncture planning and navigation system in the angiographic suite. Am. J. Neuroradiol. 35, 2170–2175. 10.3174/ajnr.A4009 24994826 PMC7965169

[B15] Zhang C. Gao H. Liu Z. Huang H. (2021). The potential value of mixed reality in neurosurgery. J. Craniof. Surg. 32, 940–943. 10.1097/SCS.0000000000007317 33290332

[B16] Zheng M. Qi G. Zhu Z. Li Y. Wei H. Liu Y. (2020). Image dehazing by an artificial image fusion method based on adaptive structure decomposition. IEEE Sensors J. 20, 8062–8072. 10.1109/JSEN.2020.2981719

[B17] Zhou M. Wang H. Zeng X. Yin P. Zhu J. Chen W.. (2019). Mortality, morbidity, and risk factors in China and its provinces, 1990-2017: a systematic analysis for the global burden of disease study 2017. Lancet 394, 1145–1158. 10.1016/S0140-6736(19)30427-1 31248666 PMC6891889

[B18] Zhu Z. Wei H. Hu G. Li Y. Qi G. Mazur N. (2021). A novel fast single image dehazing algorithm based on artificial multiexposure image fusion. IEEE Trans. Instrum. Measure. 70, 1–23. 10.1109/TIM.2020.3024335

